# A Cas12a Toolbox for Rapid and Flexible Group B *Streptococcus* Genomic Editing and CRISPRi

**DOI:** 10.1111/mmi.70022

**Published:** 2025-09-13

**Authors:** G. H. Hillebrand, S. C. Carlin, E. J. Giacobe, H. A. Stephenson, J. Collins, T. A. Hooven

**Affiliations:** 1 Program in Microbiology and Immunology, University of Pittsburgh School of Medicine, Pittsburgh, Pennsylvania, USA; 2 Department of Pediatrics, Division of Newborn Medicine, University of Pittsburgh School of Medicine, Pittsburgh, Pennsylvania, USA; 3 Dietrich School of Arts and Sciences, University of Pittsburgh, Pittsburgh, Pennsylvania, USA; 4 Department of Microbiology and Immunology, School of Medicine, University of Louisville, Louisville, Kentucky, USA; 5 RK Mellon Institute for Pediatric Research, Pittsburgh, Pennsylvania, USA

**Keywords:** Cas12a, CRISPRi, genome editing, group B *Streptococcus*

## Abstract

*Streptococcus agalactiae* (group B *Streptococcus*; GBS) is a leading cause of neonatal sepsis and meningitis. Despite advances in molecular microbiology, GBS genome engineering remains laborious due to inefficient mutagenesis protocols. Here, we report a versatile and rapid Cas12a-based toolkit for GBS genetic manipulation. We developed two shuttle plasmids—pGBSedit for genome editing and pGBScrispri for inducible CRISPR interference—derived from an *Enterococcus faecium* system and optimized for GBS. Using these tools, we achieved targeted gene insertions, markerless deletions, and efficient, template-free mutagenesis via alternative end-joining repair. Furthermore, a catalytically inactive dCas12a variant enabled inducible gene silencing, with strand-specific targeting effects. The system demonstrated broad applicability across multiple GBS strains and minimal off-target activity, as confirmed by whole-genome sequencing. In benchmarking, template-less Cas12a mutagenesis yielded sequence-confirmed mutants in ~7 days and homology-directed edits in ~7–14 days; aTC-resistant colonies arose at ~10^−4^ of uninduced CFU, and 27%–65% of resistant clones carried the intended homology-directed edit depending on locus and homology arm length (e.g., ~27% markerless deletion; ~35% insertion; 65% with 1 kb arms). These workflows provide a rapid alternative to temperature-sensitive plasmid mutagenesis protocols that typically require ≥ 4 weeks. This Cas12a-based platform offers an efficient, flexible, and scalable approach to genetic studies in GBS, facilitating functional genomics and accelerating pathogenesis research.

## 1 ∣ Background

*Streptococcus agalactiae* (group B *Streptococcus*; GBS) is a cause of severe infections, particularly among perinatal populations: pregnant women, newborns, and infants (Lawn et al. 2017; Gonçalves et al. 2022). Genetic research is key to the development of GBS vaccines and targeted therapeutics (Patras and Nizet 2018; Brokaw et al. 2021; Kobayashi et al. 2016).

Currently, most protocols for GBS mutagenesis are based on temperature-dependent shuttle plasmids (Yim and Rubens 1998; Hooven et al. 2019). The process for using a temperature-sensitive mutagenesis plasmid begins with recombinant insertion of an editing cassette with flanking GBS homologous sequences. After transformation into GBS, targeted plasmid integration into the circular chromosome can be promoted by transitioning the culture to a temperature that restricts extrachromosomal plasmid replication (Yim and Rubens 1998; Framson et al. 1997). In a subsequent self-excision step, the targeted region of the chromosome undergoes a second recombination event in which the native sequence is replaced by the mutagenesis cassette originally introduced on the plasmid (Hooven et al. 2019).

While temperature-sensitive plasmids have been used to generate many GBS mutants, successful use of these techniques remains time-consuming and laborious. One drawback is that—to propagate the mutagenesis plasmid during the initial cloning and transformant outgrowth steps—the cultures must be maintained at low temperature (usually 28°C), which slows bacterial growth and prolongs the experimental timeframe. Another problem is that the multiple passaging and outgrowth steps create opportunities for unintended recombination and spontaneous mutation to occur. The result is that, even when using a counterselection-optimized mutagenesis plasmid, it can take months to create a single targeted GBS mutation. When un-expected problems arise, often the only remedy is to return to the first step of the process, which delays progress.

Mutagenesis approaches based on the activity of clustered regularly interspaced short palindromic repeats and CRISPR-associated protein (CRISPR/Cas) have revolutionized genetic and molecular biology research (Mali et al. 2013; Doudna and Charpentier 2014; Jinek et al. 2012). Multiple CRISPR/Cas system variants evolved in bacteria as defense mechanisms against exogenous, bacteriophage-encoded DNA (Chen and Doudna 2017; Murugan et al. 2017). Bioengineered CRISPR/Cas systems have become useful as easily programmable, targeted molecular effectors of numerous experimental functions including flexible genome editing (Pickar-Oliver and Gersbach 2019).

Techniques for using CRISPR/Cas for bacterial genome editing have proven effective (Kurushima and Tomita 2022; Choi and Lee 2016; Oh and van Pijkeren 2014; Jiang et al. 2017). However, these efforts can be complicated by the presence of one or more native CRISPR/Cas systems encoded on the genome of the study species. In 2023, we introduced a system for using a catalytically inactive dCas9 variant, encoded on the chromosome of bioengineered mutant GBS strains, to downregulate gene expression through CRISPR interference (CRISPRi) (Gopalakrishna et al. 2023). We also made multiple unreported, unsuccessful efforts to use the native GBS CRISPR/Cas system to drive an effective genome editing approach.

An alternative approach to bacterial genome editing is through the introduction of non-native bioengineered CRISPR/Cas systems. Cas12a (also referred to as Cpf1) is a type V-A endonuclease found in class 2 CRISPR/Cas systems. Cas12a performs programmable targeting of specific DNA sequences through a complex between the Cas12a enzyme and a CRISPR RNA (crRNA) strand made up of a target-complementary spacer sequence and a conserved direct repeat sequence (Jiang et al. 2017; Senthilnathan et al. 2023; Zetsche et al. 2015; Yan et al. 2017; Meliawati et al. 2021). Unlike Cas9, Cas12a does not require the presence of a trans-activating CRISPR RNA (tracrRNA) sequence for programmable targeting of specific DNA sequences (Zetsche et al. 2015). This allows for simpler plasmid-based platforms for Cas12a introduction into new experimental systems. Cas12a requires a T-rich protospacer-adjacent motif (PAM) to cleave a target sequence, which makes it an appealing choice for use in low-GC organisms such as GBS (Zetsche et al. 2015).

Recent successful development of a Cas12a-based system for genome editing in *Enterococcus faecalis* prompted us to examine its potential use in GBS (Chua and Collins 2022). Here we report development, testing, and optimization of a single-plasmid Cas12a-based suite of GBS genetic tools. We have used our Cas12a system for allelic exchange mutagenesis, alternative end-joining mutagenesis, and targeted CRISPRi gene knockdown using a catalytically deactivated Cas12a (dCas12a) variant. Our strategy is efficient. Using it, we have made numerous mutants in an approximately one-week timeframe. Another advantage over other GBS genetic tools (such as our dCas9-based CRISPRi technique) is that it can be used across different GBS strain lineages with no requirement for preceding alterations to the native chromosome. In summary, we find that using Cas12a for site-directed GBS mutagenesis and genetic expression modulation is a significant improvement over previously described techniques.

## 2 ∣ Results

### 2.1 ∣ Chromosomal Insertion of an eGFP Expression Cassette Using Cas12a and Homology-Directed Repair

**FIGURE 1 ∣:**
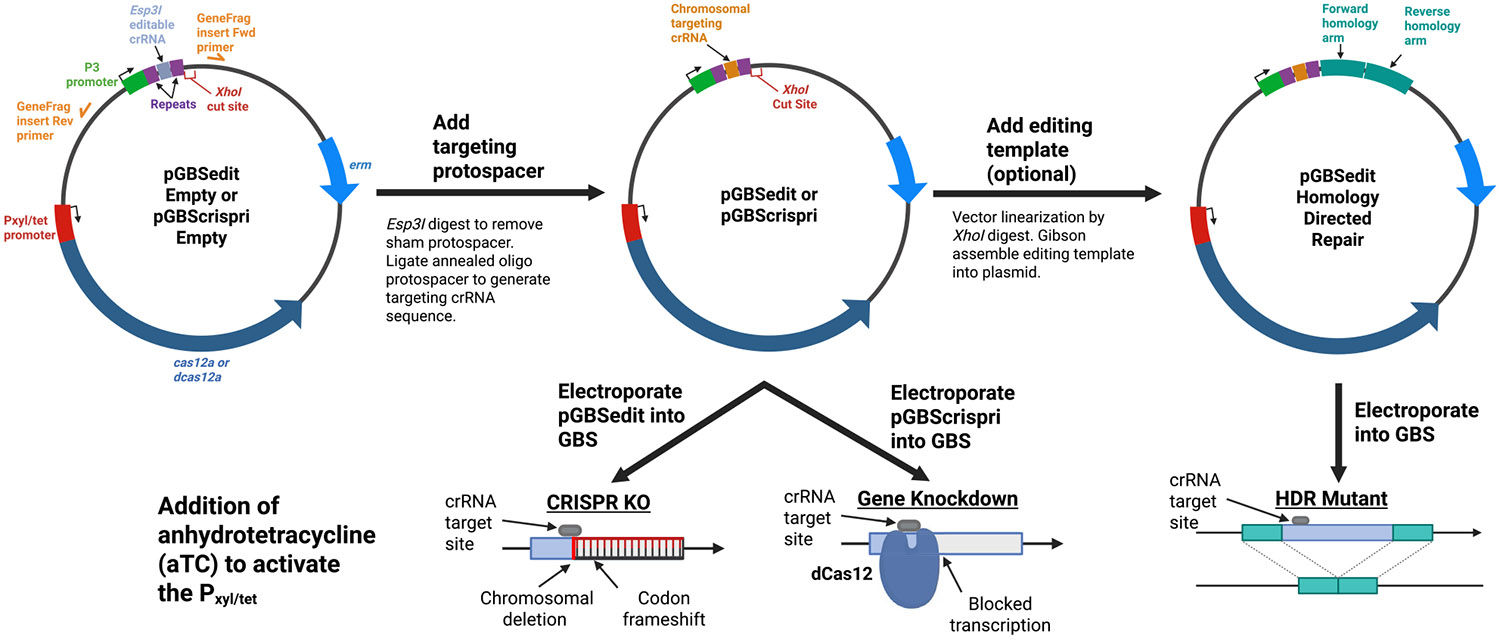
Design and workflow of the Cas12a-based GBS genetic toolkit. Plasmid maps and workflows of the Cas12a-based genetic toolkit for Group B *Streptococcus*. The two shuttle vectors—pGBSedit (encoding wild type Cas12a) and pGBScrispri (encoding catalytically inactive dCas12a)—carry a Pxyl/tet-inducible promoter upstream of the cas12a/dcas12a gene, a constitutive TetR repressor, and an *Esp3I*-flanked spacer insertion site; pGBSedit also includes a unique *XhoI* site for cloning homology arms. Three experimental protocols are illustrated: (1) template-less mutagenesis, where Cas12a-induced double-strand breaks are repaired via the native alternative end-joining pathway to generate variable indels; (2) inducible CRISPR interference, in which dCas12a binding to the target locus upon anhydrotetracycline (aTC) induction leads to targeted gene knockdown; and (3) homology-directed repair mutagenesis, in which an editing template directs precise genomic insertions or deletions followed by aTC selection.

Figure 1 diagrams the design and potential uses of the two plasmids that underpin our suite of Cas12a-based GBS genetic tools. pGBSedit is designed for genome editing, while pGBScrispri is for CRISPRi. Both plasmids are modified versions of the Cas12a-based shuttle plasmid pJC005, which was previously developed for *E. faecium* genetic modification (Chua and Collins 2022). pGBSedit and pGBScrispri both encode conserved gram-negative and gram-positive origins of replication so that CRISPR RNA (crRNA) and homology arm cloning can be performed in *Escherichia coli* followed by electroporation transformation of purified plasmid into GBS.

pGBSedit encodes wild-type (WT) *Acidaminococcus* Cas12a whereas pGBScrispri encodes a modified, catalytically inactive (E993A) dCas12a that can be used for inducible, targeted gene repression. In both versions of the plasmid, the *cas12a*/*dcas12a* gene is downstream of a Pxyl/tet inducible promoter bioengineered to maximize the dynamic range in response to its anhydrotetracycline (aTC) inducer, with minimal expression in the uninduced state and strong expression upon aTC exposure (Helle et al. 2011). TetR, the promoter's repressor, is constitutively expressed on the plasmid.

The crRNA cassette is identical in pGBSedit and pGBScrispri. Driven by a constitutive P3 small RNA-optimized promoter, the crRNA region harbors a pair of apposed *Esp3I* restriction enzyme cut sites that, when digested, yield incompatible sticky ends into which a custom dsDNA spacer sequence can be ligated to encode a functional crRNA.

Once an active crRNA is established, with genomic target site complementarity adjacent to a Cas12a PAM, aTC induction of WT *cas12a* from pGBSedit leads to reliable selection against all GBS cells bearing the target sequence-PAM combination. Viable CFUs are enriched for variants that escaped selection by mutation of the target site.

One experimentally useful mechanism of chromosomal repair is homologous recombination with an editing template that can be cloned onto the pGBSedit plasmid at a *XhoI* restriction enzyme recognition site downstream of the crRNA cassette. To demonstrate the effectiveness of this approach, we created an editing template in which the *eGFP* gene and a P23 constitutive promoter are flanked by approximately 500-nt homology arms that direct it to an intergenic region conserved in CNCTC 10/84 and A909 GBS strains.

**FIGURE 2 ∣:**
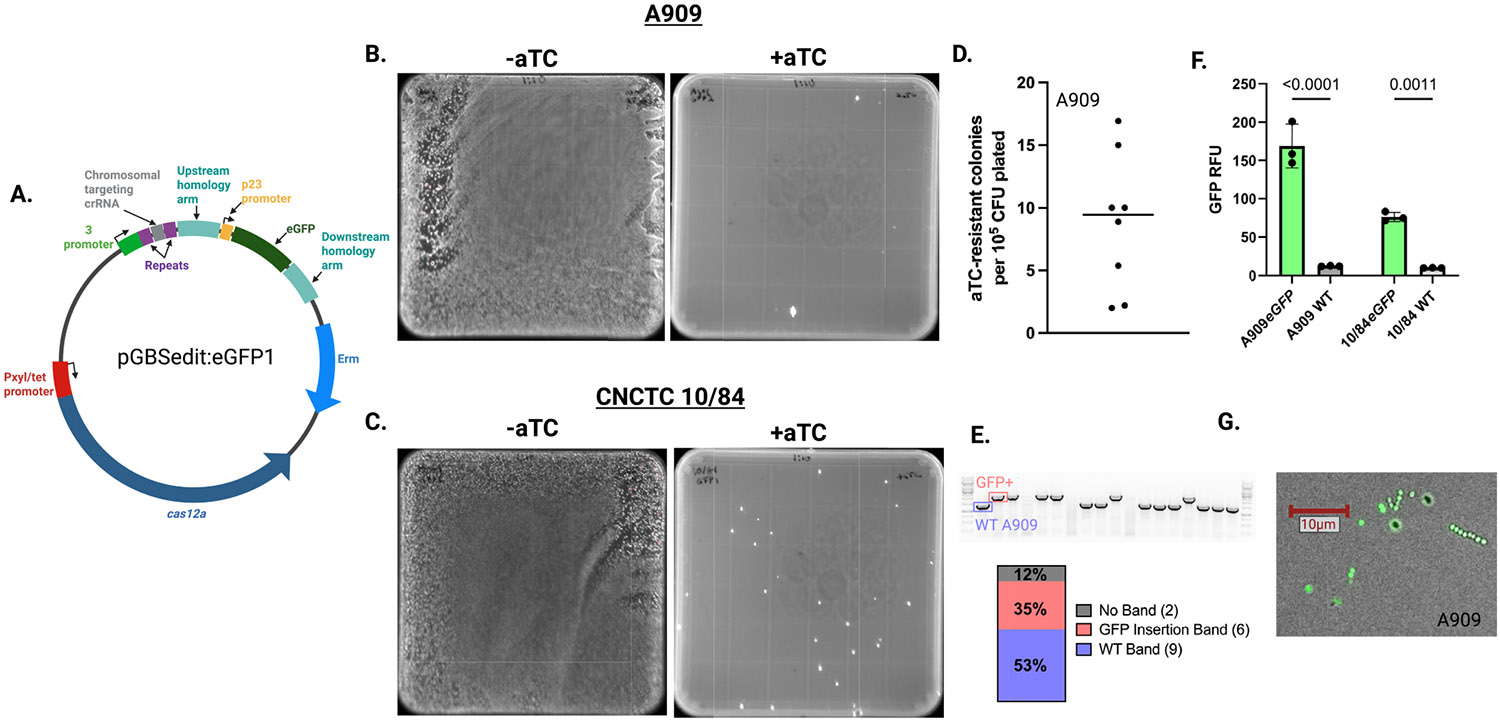
Cas12a-mediated insertion of eGFP into the GBS chromosome via homology-directed repair. (A) Diagram of pGBSedit:EGFP1, with ~500-nt upstream and downstream homology arms flanking the *eGFP* coding sequence targeted to an intergenic locus conserved in A909 and CNCTC 10/84. (B, C) Representative agar plates of A909 (B) and CNCTC 10/84 (C) transformants on erythromycin: Without aTC (left) showing unselected background, and with 500 ng/mL aTC (right) showing only *eGFP* integrants. (D) Quantification of aTC-resistant colonies obtained in A909 following transformation with pGBSedit:EGFP1. Each point represents an independent transformation; the line marks the mean (9.4 × 10^−5^). (E) Representative colony PCR genotyping of A909 aTC-resistant colonies. Gel image shows expected WT band (blue) and GFP insertion band (red), with proportions of each genotype indicated below. (F) Fluorescence quantification (*n* = 3; mean ± SD, Student's *t*-test). (G) Imaging of genome edited A909 under blue-light illumination confirming *eGFP* expression in resuspended colonies.

After cloning this editing template into pGBSedit, along with a targeting crRNA directed to the intended genomic insertion site (generating pGBSedit:eGFP1, Figure 2A), we transformed WT CNCTC 10/84 and A909 with the targeting plasmid. In the uninduced state without aTC, a liquid culture of the transformed GBS strain grew as an unselected lawn on solid agar media (Figure 2B,C, left side). However, when exposed to aTC added atop the agar media, there is dramatic and reliable selection against the WT background that comprises most of the lawn (Figure 2B,C, right side). In eight independent A909 biological replicates quantified for selection efficiency, the mean rate of aTC resistance was 9.4 × 10^−5^ (Figure 2D) Among the aTC-resistant colonies, a substantial fraction carried the intended eGFP insertion as confirmed by colony PCR (Figure 2E) and expressed eGFP fluorescence (Figure 2F,G).

### 2.2 ∣ Markerless Gene Deletion Using Cas12a Homology-Directed Repair

An anticipated use of the Cas12a tools in GBS is markerless gene (or other genomic region) deletion. To demonstrate the feasibility of this application and to quantify various possible Cas12a selection endpoints, we created pGBSedit:Δ*covR1* to create a markerless deletion of the *covR* gene. CovR is part of the two-component signaling system that regulates numerous GBS genes (Mazzuoli et al. 2021; Claverie et al. 2024), including the *cyl* operon responsible for the biosynthetic pathway of β-hemolysin/cytolysin (βHC), a pigmented, hemolytic GBS toxin (Armistead et al. 2020; Randis et al. 2014). We chose this target gene because its deletion leads to *cyl* de-repression and an easily assayed hyperpigmented, hyperhemolytic phenotype (Lupo et al. 2014).

**FIGURE 3 ∣:**
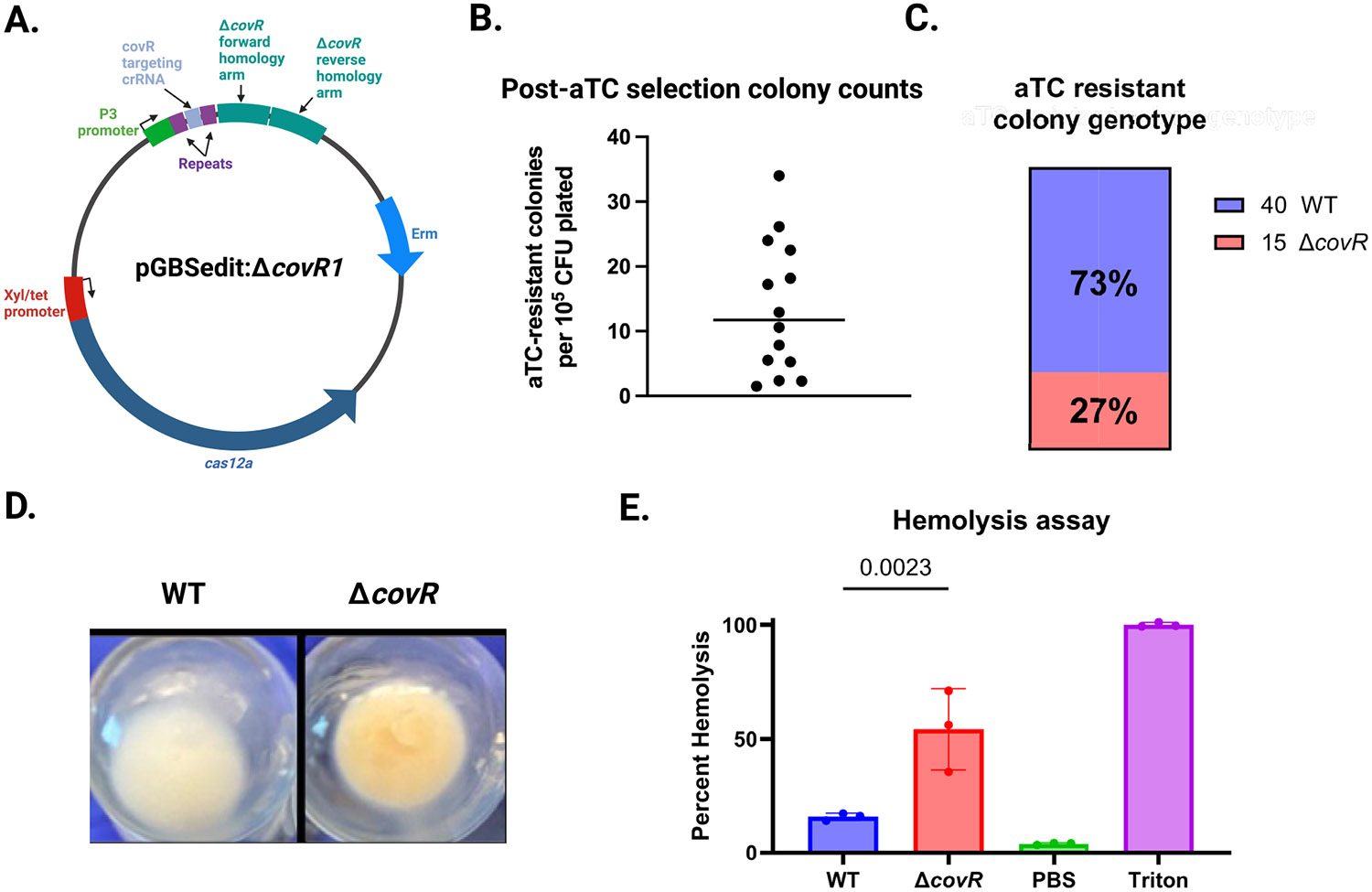
Markerless deletion of covR using Cas12a homology-directed repair. (A) Design of pGBSedit:ΔcovR1, containing fused ~500 bp homology arms upstream and downstream of the *covR* coding sequence. (B) Selection resistance frequency for Δ*covR1* across 14 independent aTC selections in A909; each symbol represents one biological replicate, and the horizontal bar denotes the mean (1.1 × 10^−4^). (C) Genotyping by colony PCR of 55 aTC-selected colonies from eight biological replicates; 27% yielded the expected Δ*covR* amplicon. (D) Photograph of liquid cultures showing hyperpigmented phenotype of confirmed Δ*covR* mutants. (E) Quantification of hemolytic activity for Δ*covR* versus WT, measured by released hemoglobin absorbance (*n* = 3; mean ± SD, Student's *t*-test).

pGBSedit:Δ*covR1* has an anti-*covR* targeting crRNA directed to the target sequence next to the 3′-TTTC PAM at nucleotide position 436 in the *covR* gene. In the pGBSedit *XhoI* site, we cloned an approximately 1000-bp editing template composed of fused 500-bp *covR* upstream and downstream homology arms (Figure 3A).

After transformation of purified pGBSedit:Δ*covR1* into WT A909, we performed 14 independent aTC selection experiments during which we quantified the unselected background CFU concentration and the aTC selection output. The mean rate of selection escape across our 14 biological replicates was 1.1 × 10^−4^ (range 1 × 10^−5^—3.5 × 10^−4^, Figure 3B). We performed colony PCR on colonies from 8 independent biological replicates of the transformation step. Among the 55 PCR reactions that generated an electrophoresis gel band, 15 (27%) were of the expected size for the in-frame gene deletion, while the remaining were consistent with wild type (Figure 3C). Inspection of the 15 suspected knockouts showed that all had the expected hyperpigmented appearance when grown in liquid culture (Figure 3D). To confirm that the knockout strain was hyperhemolytic, we performed three biological replicates of hemolysis assays with one of the genotypic knockouts from our experiment, which confirmed the expected phenotype (Figure 3E).

To understand the influence of editing template homology arm length on Cas12a markerless chromosomal deletion rates, we performed an experiment in which we created a partial deletion of the *eGFP* gene in the A909*eGFP* strain created as described above, using two different lengths of homology arms, 500 and 1000 bp. The crRNA targeting site in this experiment was located downstream of *eGFP*, outside of the open reading frame, so that alternative end-joining events (see below section) would not result in a phenotypic knockout. Only template-based editing, from the homology arms whose union deletes the promoter and first approximately 20% of *eGFP*, would result in phenotypic loss of fluorescence. This design allowed us to examine phenotype to surmise genotype. It revealed that the 1000-bp homology arms were more efficient than the 500-bp homology arms, leading to a 65% versus 39% success rate (Figure S1). This difference must be weighed against diminishing transformation rates with pGBSedit bearing longer homology arms, and the potentially increased cost and time requirements of bioengineering longer homology arm editing cassettes.

### 2.3 ∣ CRISPR/Cas12a GBS Mutagenesis by Alternative End-Joining

**FIGURE 4 ∣:**
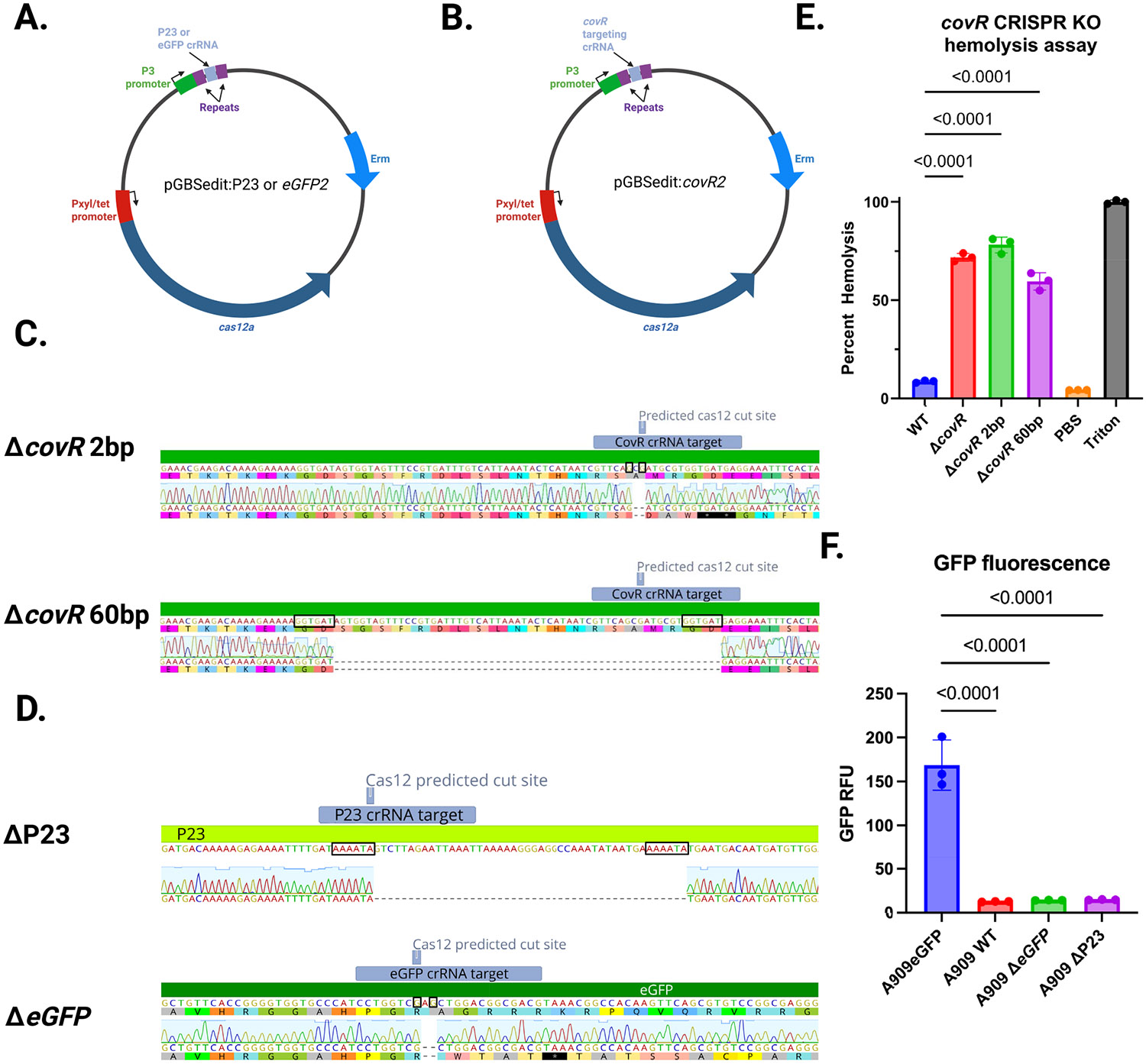
CRISPR/Cas12a-mediated free end-joining mutagenesis in GBS. (A, B) Plasmid designs for template-less mutagenesis: PGBSedit:CovR2, pGBSedit:EGFP2, and pGBSedit:P23, each encoding a crRNA targeting covR, chromosomal eGFP, or the P23 promoter. (C) Representative sequence alignments from covR2 mutants showing a 2-bp deletion and a 60-bp deletion at the target site. Microhomology regions suspected to have served as loci for alternative end-joining are boxed. (D) Indel patterns at the P23 and eGFP spacer sites in selected mutants. Microhomology regions suspected to have served as loci for alternative end-joining are boxed. (E) Hyperhemolytic phenotype of *covR* mutants in a hemolysis assay (*n* = 3; mean ± SD, ANOVA with Dunnett's correction). (F) Loss of eGFP fluorescence in *eGFP2* mutants and reduced P23 promoter activity in P23 mutants (*n* = 3; mean ± SD, ANOVA with Dunnett's correction).

To examine whether Cas12a-mediated mutations at the genomic target site occur in the absence of homologous recombination with an editing cassette, we generated *covR* and *eGFP* targeting versions of pGBSedit without homology-based editing templates. pGBSedit:*covR2* and pGBSedit:*eGFP2* target the *covR* and *eGFP* coding sequences, respectively; pGBSedit:P23 targets the P23 promoter upstream of *eGFP* (Figure 4A,B). We transformed WT A909 with pGBSedit:*covR2* and A909 with chromosomal eGFP (A909eGFP) with pGBSedit:*eGFP2* or pGBSedit:P23. After selection with aTC, we examined the surviving colonies for phenotypic changes, noting that the majority of the WT A909 + pGBSedit:*covR2* colonies were hyperpigmented and hyperhemolytic, and the A909eGFP + pGBSedit:*eGFP2* and A909eGFP + pGBSedit:P23 colonies were not fluorescent. We performed colony PCR of the genomic regions flanking the three Cas12a target sites, then Sanger sequenced the resulting bands. This analysis identified two genomic deletions surrounding the Cas12a targeted sites in the *covR* targeted colonies (Figure 4C). One deletion was 2 bp in size and the other was 60 bp. Sanger sequencing revealed one genomic deletion each for the P23 promoter and *eGFP* targeted strains (Figure 4D). Predictably, mutations that delete significant portions of the P23 promoter and coding sequence frameshift mutations lead to loss of gene function in phenotypic testing (Figure 4E,F).

While the mutations that emerge from this template-less CRISPR editing approach are unpredictably sized, we find that the speed and ease with which they can be generated make this an attractive protocol for rapid creation of mutants in regions of interest. In our benchmarking experiments, the full pipeline of crRNA cloning, GBS transformation and aTC selection, and PCR/Sanger sequence confirmation could all be completed in a week. Promising genomic changes made through this approach can then be more rigorously verified by markerless deletion or allelic exchange with an editing template.

### 2.4 ∣ Whole-Genome Sequencing of Cas12a-Edited GBS Genomes Does Not Indicate Substantial Off-Target Activity

A persistent concern with CRISPR-based genomic editing is the potential for off-target mutagenesis. Cas12a editing systems have been shown to have promiscuous nickase activity (Murugan et al. 2020) that could drive higher than baseline rates of mutation if erroneous repair mechanisms were widely activated. To investigate this possibility, we performed Illumina whole-genome sequencing of mutant strains recovered and tested in the above experiments. After unbiased de novo alignment and contig scaffolding against a reference GBS genome, we identified between 1 and 5 SNPs across our sequenced mutant strain genomes (Figure S2). Additionally, a prophage resident in the A909 genome was observed to have excised in one of our sequenced strains, which we have encountered in previous studies unrelated to Cas12a mutagenesis (Gopalakrishna et al. 2023). Given estimated secular mutation rates in GBS of approximately 10^−9^ mutations per bp per generation (Drake et al. 1998; Cuny et al. 2021; Rosinski-Chupin et al. 2013; Sheppard et al. 2018), or approximately 0.002 mutations per 2 M-bp genome per generation, and roughly 20 generations per laboratory outgrowth, 1–5 fixed SNPs in a strain that is 5–10 outgrowth steps from its progenitor roughly matches expectations and does not indicate widespread or biased off-site mutagenesis activity by Cas12a.

### 2.5 ∣ Inducible Gene Silencing With dCas12a

**FIGURE 5 ∣:**
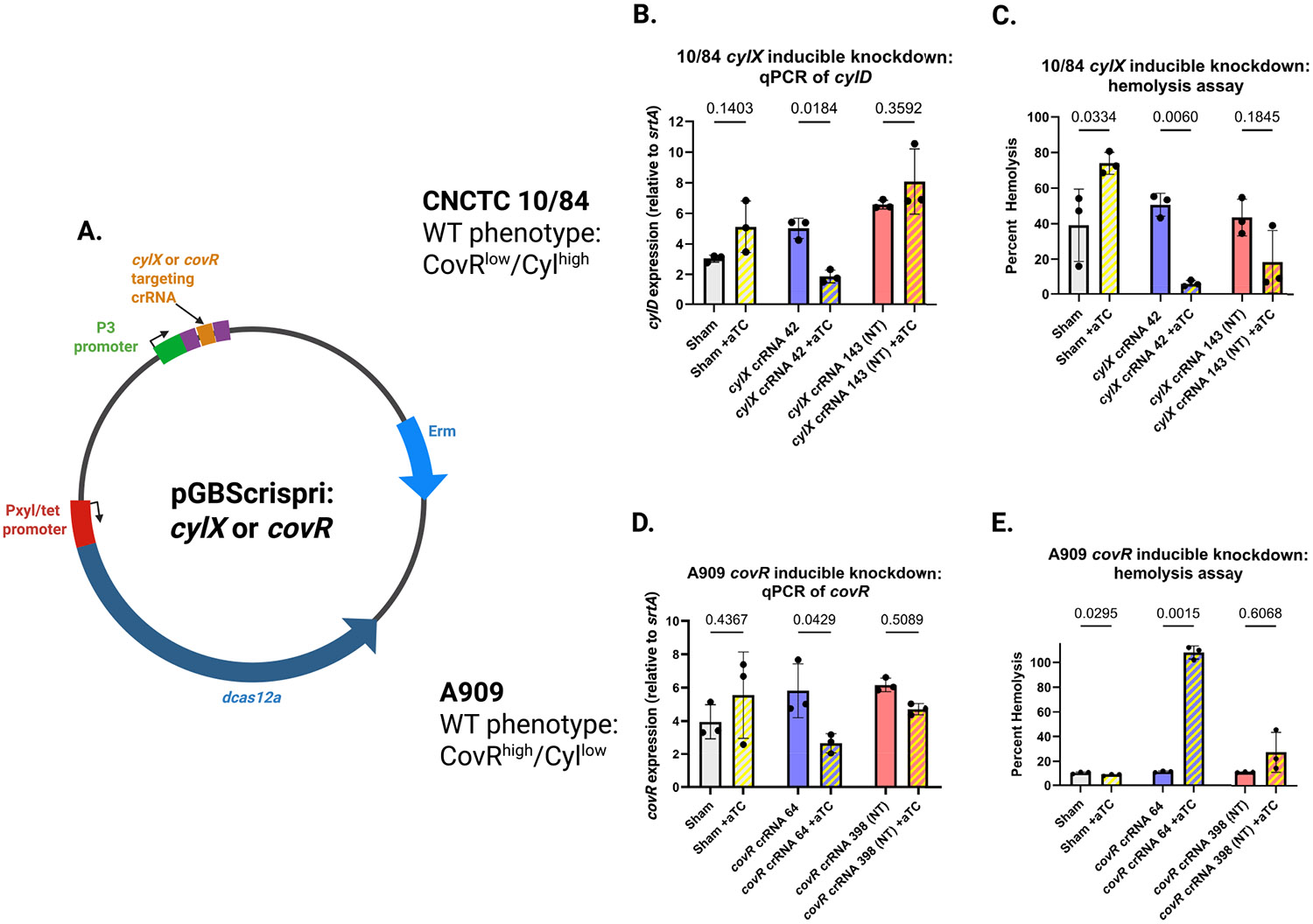
Inducible gene silencing in GBS using dCas12a CRISPR interference. (A) Plasmid designs for dCas12a targeting: *cylx*42 (template strand) and *cylx*143 (non-template strand) in the *cyl* operon, or *covR*64 (template strand), *covR*398 (non-template strand) in *covR*. (B) qRT-PCR quantification of *cyl* expression in CNCTC 10/84 with (+ aTC) or without (− aTC) induction (*n* = 3; mean ± SD, ANOVA with correction for multiple comparisons), showing stronger knockdown with template-strand targeting. mRNA measurements were normalized to *srtA* expression. (C) Hemolysis assays of CNCTC 10/84 strains demonstrating reduced βHC activity upon dCas12a targeting of *cyl* (*n* = 3; mean ± SD, ANOVA with correction for multiple comparisons). (D) qRT-PCR of *cyl* expression in A909 harboring *covR*-targeting dCas12a, showing de-repression upon induction (*n* = 3; mean ± SD, ANOVA with correction for multiple comparisons). mRNA measurements were normalized to *srtA* expression. (E) Hemolysis assays of A909 *covR* knockdown strain confirming increased βHC activity with aTC induction (*n* = 3; mean ± SD, ANOVA with correction for multiple comparisons). Throughout, sham = pGBScrispri with a random, non-targeting crRNA without homology to the GBS genome.

To explore the potential of using dCas12a as a tool for inducible gene silencing, we used crRNA targeting βHC biosynthetic enzymes encoded by the *cyl* operon and crRNA targeting *covR* (Figure 5A). Previous studies of dCas12a-based gene silencing have demonstrated strand bias in which crRNA targeting the template strand of the gene has a stronger silencing effect than crRNA targeting the non-template strand (Zhang et al. 2017). This is the opposite of dCas9 gene silencing, which works best when the non-template strand is targeted by the guide RNA (dCas9 template strand targeting can actually increase gene expression in some cases) (Gopalakrishna et al. 2023; Qi et al. 2013). To examine this aspect in our system, we used both template and non-template targeting crRNA.

We transformed CNCTC 10/84 with pGBScrispri:*cylx42* (template targeting) and pGBScrispri:*cylx143* (non-template targeting). CNCTC 10/84 is a naturally hyperpigmented/hyper-hemolytic GBS strain, due to a SNP in the *covR* promoter that results in constitutively low expression (Zhu et al. 2020), so suppressing *cyl* in this background is expected to reduce pigmentation and cytotoxicity. By qRT-PCR (Figure 5B) and in vitro hemolysis assays (Figure 5C), we observed *cyl* downregulation in the presence of the aTC inducer and a targeting crRNA. Addition of aTC without a targeting spacer was noted to cause a small but consistent upregulation of *cyl* expression, both by qRT-PCR and phenotypic assay. As previously described, the template strand targeting pGBScrispri:*cylx42* plasmid led to greater gene repression than the non-template targeting pGBScrispri:-*cylx143* variant.

Silencing *covR* in A909, which is not a naturally hyperhemolytic strain, is expected to cause *cyl* de-repression and overexpression of the βHC toxin. Again, this expected result was observed by qRT-PCR (Figure 5D) and hemolysis assays (Figure 5E), only in the presence of the aTC inducer. (In contrast to CNCTC 10/84, aTC did not measurably increase βHC expression in A909 when no *covR* targeting crRNA was present, see Figure 5E leftmost two columns). Together, these results demonstrate the utility of inducible dCas12a as a rapid and reliable tool for targeted gene expression knockdown in GBS.

## 3 ∣ Discussion

We have described a suite of Cas12a-based molecular tools to facilitate genetic experiments in GBS. While Cas9-based platforms have dominated the first decade of widespread CRISPR/Cas-mediated biology, other Cas variants have been found to be superior in some circumstances. The type V-A Cas12a enzyme, variants of which were first characterized in *Acidaminococcus* spp. and *Lachnospiraceae* spp., generates staggered DNA cleavage ends 18 nt (on the crRNA homologous strand) and 23 nt (on the crRNA complementary strand) from the PAM (Zetsche et al. 2015). This is different from Cas9, which generates blunt-end double-stranded DNA cleavage (Chen and Doudna 2017). Also distinct from Cas9 are the location and sequence requirements of the Cas12a PAM. Whereas Cas9 uses a 5′ PAM, which in most *Streptococci* has a 5′-NGG sequence requirement, Cas12a recognizes 3′-TTTV upstream of the target sequence.

A major advantage of Cas12a-based technology for altering the GBS genome or gene is that, because of significant structural differences between Cas12a and Cas9, the native GBS CRISPR/Cas9 system has no crosstalk with exogenously introduced Cas12a molecules. This is especially relevant to pathogenesis research, given recent evidence that Cas9 expression is itself modulated in GBS in ways that intersect significantly with virulence regulation (Pastuszka et al. 2025). The resources described here should allow investigators to choose from several complementary approaches for GBS chromosomal manipulation in mechanistic studies of GBS colonization and infection.

The dCas12a-based CRISPRi system is effective for inducible targeted gene knockdown, as demonstrated by our successful downregulation of the *cyl* operon and the *covR* gene that encodes a key transcriptional regulator of *cyl* and its expression of the hemolytic toxin βHC. In experiments with our recently published dCas9-based CRISPRi system, we have shown that CRISPRi efficacy is variable (Gopalakrishna et al. 2023). Targeting dCas9 to the 5′ end of a protein-coding gene generally has a greater knockdown effect than targeting closer to the 3′ gene terminus, but even different spacers directing dCas9 to the first half of a protein coding sequence can cause variable decreases in gene expression. We expect similar findings with dCas12a-based CRISPRi and recommend that—whenever possible—researchers examining gene function with dCas12a employ multiple different spacers targeting their gene of interest to boost the odds of maximizing the knockdown effect with one of them.

The capabilities around direct chromosomal mutagenesis afforded by our system open new possibilities in terms of speed and ease of generating new genetic variants. The protocol for using a crRNA-only plasmid construct to generate variable end-joining mutations at the target site can be completed in a week, which is a significant advance over temperature-based plasmid selection/counter-selection systems. The recombinant DNA mechanism underlying this end-joining mutagenesis phenomenon is not fully clear. CRISPR/Cas-based genome editing in eukaryotic cells can make use of non-homologous end-joining (NHEJ) mechanisms, but the enzymatic systems responsible for NHEJ are not present in bacteria. Mechanistically simpler NHEJ processes, which depend on ATP-dependent ligase-D and Ku enzymes, exist in *Mycobacteria* and *Bacillus* species, but are not encoded by GBS (Della et al. 2004; Weller et al. 2002; Shuman and Glickman 2007).

A different bacterial double-stranded break repair pathway, known as alternative end-joining (A-EJ) appears more likely to be functioning in the repair-driven mutagenesis we observe in GBS following chromosomal Cas12a targeting (Chayot et al. 2010; Hou et al. 2024). A-EJ is a process of microhomology-based DNA recombination. A-EJ is categorically based on recombination repairs of micro-deletions, and (unlike indels) does not create duplications or other sequence-expanding insertions. First described in *E. coli*, A-EJ of bacterial chromosomal breaks is partially dependent on the RecBCD complex driving free end degradation, leading eventually to exposure of compatible microhomologous sequences (approximately 3-nt on average) that drive initial re-synapsis of apposing ends, followed by gap repair. Because of the initial bidirectional free end degradation that underpins A-EJ, the mutations left behind in the process reveal short or long sequence deletions eventually resolved by stochastic microhomology-driven reannealing of the two free ends. Small single-stranded gaps left over after microhomology synapsis are suspected to be repaired by the essential enzyme LigA, followed by resolution of ssDNA flaps by 3′ exonucleases or SbcCD (Lee et al. 2021). Because targeted Cas12a-initiated A-EJ creates variable, often frameshifted chromosomal deletions, it can be harnessed as a tool for bioengineering experimental mutations in GBS, as we have shown here. A predicted mechanism for GBS A-EJ is diagrammed in Figure S3.

Cas12a selection against WT chromosomal sequences can also be used to screen for allelic exchange events generated by homology-driven recombination with an editing template cloned into the same pGBSedit plasmid that carries the Cas12a and crRNA coding sequences. This approach mirrors widely used CRISPR/Cas genome editing strategies that have been effectively harnessed for studies on organisms across the tree of life. We have successfully used Cas12a allelic exchange mutagenesis to replace genes with antibiotic resistance markers, generate markerless gene deletions and SNPs, and insert new sequences at specific sites on the chromosome.

This work highlights the value of flexible tool design when working in genetically diverse species like GBS. The pGBSedit and pGBScrispri plasmids were constructed with modular components that facilitate rapid adaptation to different experimental aims, whether targeting chromosomal loci for allelic exchange or downregulating gene expression through inducible repression. Because these tools do not rely on prior genomic alterations, they are well suited to applications across strain backgrounds and experimental conditions.

Although this system streamlines many aspects of mutagenesis and gene regulation in GBS, certain limitations remain. As with other CRISPR-based systems, off-target effects cannot be entirely ruled out. Our tetracycline-inducible Cas12a system allows suppression of the enzyme until a temporary exposure drives selection against the target (WT) genetic sequence, limiting the duration of susceptibility to Cas12a off-target effects. Accordingly, our whole-genome sequencing results for the mutants we generated in this study did not suggest indiscriminate mutagenesis from widespread Cas12a nickase activity. However, the possibility of unintended mutations remains; whole-genome sequencing or complementary genetic controls are therefore advisable when feasible. In addition, while the ease of using end-joining mutagenesis is appealing, its reliance on stochastic microhomology limits its precision, and it may not be appropriate for applications requiring tightly defined genomic edits.

In summary, the Cas12a-based genetic toolkit presented here provides a set of versatile, efficient alternatives to more cumber-some traditional methods for GBS manipulation. By supporting targeted mutagenesis, promoter knockdown, and recombination-driven genome editing, these tools may facilitate broader efforts to interrogate GBS gene function and pathogenesis. We anticipate that their continued refinement and use will help advance genetic research in this clinically important organism.

## 4 ∣ Methods and Materials

### 4.1 ∣ Ethics Statement

Whole blood collection by phlebotomy of healthy adult volunteers was conducted in accordance with an approved University of Pittsburgh IRB protocol (CR19110106-001). Following the use of blood in GBS hemolysis assays, samples were discarded and no further volunteer-level data were collected.

### 4.2 ∣ Reagents

LB liquid broth mix, LB agar, TS liquid broth mix, TS agar, erythromycin (Erm), anhydrotetracycline, M17 liquid medium mix, glycine, *Esp3I*, T4 ligase, T4 polynucleotide kinase, *XhoI*, Gibson assembly master mix, *DpnI*, Q5 Taq polymerase, Qiagen QIAprep Spin Miniprep Kit, M17 broth, PEG6000.

### 4.3 ∣ Bacterial Strains and Growth Conditions

GBS strains A909 (serotype Ia, sequence type 7) and CNCTC 10/84 (serotype V, sequence type 26) (Zhu et al. 2020; Nizet et al. 1996; Lancefield et al. 1975) and their derivatives were grown at 37°C under stationary conditions in tryptic soy (TS) medium (Fisher Scientific cat. # DF0370-17-d). GBS was supplemented with 5 μg/mL Erm as needed for selection. *E. coli* strain DH5α was grown at 37°C with shaking in Luria-Bertani (LB) medium (Fisher Scientific cat. # DF9446-07-5) supplemented with 300 μg/mL Erm as needed for selection.

### 4.4 ∣ Statistical Analyses

Statistical analyses were performed with GraphPad Prism v.10.2.3 for macOS.

### 4.5 ∣ Construction of Plasmids

Plasmid backbone pJC005 (Chua and Collins 2022) was used as a backbone for pGBSedit and pGBScrispri. A modular crRNA expression cassette with a small RNA promoter (P3) driving the expression of the repeats and *Esp3I* editable sham spacer was added to both vectors. Digestion with *Esp3I* allows for complete removal of the sham spacer and ligation of a new spacer as annealed DNA oligos with matching sticky ends (pro-tospacer). A unique *XhoI* cut site was also added at the end of this cassette for linearization of the plasmid in use with Gibson Assembly addition of homology arms. This modular cloning cassette was designed and ordered as a gene fragment from Twist Bioscience, then added to both vectors via Gibson assembly. For plasmid-based complementation or overexpression, we used the pGBScomp vectors (High/Low expression; Addgene, see Supplemental Protocol Data S1 “Complementing KO strains with pGBScomp”), which accept ORFs by SalI linearization and Gibson assembly. The expression difference is from the promoter sequence. pGBScomp High has a P23 promoter and pGBScomp Low has a P_spac_ promoter upstream of the gene insertion site.

### 4.6 ∣ Protospacer Design and Insertion

Cas12a-compatible GBS genomic target sites with TTTV PAM sequences were identified with the aid of the CRISPick server hosted by the Broad Institute (Pedrozo et al. 2022). For dCas12a CRISPRi, spacer sequences complementary to the plus-strand should be selected; for Cas12a mutagenesis, plus- or minus-strand spacer sequences are equally effective. Also see Figure S4.

After selecting a spacer, the 23-nt sequence can be used to design custom ssDNA oligonucleotides for annealing to make a dsDNA protospacer, which is then cloned into *Esp3I*-digested and gel-purified pGBSedit or pGBScrispri.

### 4.7 ∣ Editing Template Design and Insertion

Editing templates included homology arms with approximately 500–1000 bp of overlap (on each end of the intended deletion or insertion). The Cas12a target site, determined with the aid of CRISPick, was positioned roughly equidistant from the homology arms. Editing constructs were ordered as synthesized gene fragments but could alternatively be generated through recombinant techniques depending on the intended end use. We cloned the editing construct into linearized *XhoI*-digested pGBSedit using Gibson assembly and used PCR and Sanger or whole-plasmid sequencing techniques to verify the insert.

### 4.8 ∣ Plasmid Purification and Transformation Into GBS

Once cloning products had been sequence-confirmed in *E. coli*, plasmids were purified using the Qiagen QIAprep Spin Miniprep Kit according to manufacturer instructions. Electrocompetent GBS was prepared and transformed as previously described (Hooven et al. 2019).

### 4.9 ∣ Outgrowth and Selection for Homology-Driven Recombination Mutants

Following GBS transformation with an editing plasmid, a single colony was resuspended in 20 μL phosphate buffered saline (PBS). 2 μL of this resuspension was used as a template for a colony PCR reaction to confirm the presence of the intended plasmid. Simultaneously, the remaining 18 μL of the PBS-resuspended sample was seeded into a 10 mL TS + Erm culture, which was allowed to grow stationary at 37°C for 6–8 h.

Following this 6–8-h outgrowth, 1:10 and 1:100 dilutions of the culture were prepared (diluted in TS + Erm). 150 μL of the following cultures were then plated on separate TS + Erm agar plates: undiluted, 1:10, and 1:100, undiluted + aTC, 1:10 + aTC, and 1:100 + aTC. For the aTC-containing cultures for plating, we add 6 μL of a 2 mg/mL aTC stock to the 150 μL of sample being plated.

These plates were allowed to grow for 36 h at 37°C. A multiple-log fold difference between colony counts on the aTC and non-aTC plates after growth is indicative of successful Cas12a selection against wild type GBS. Candidate colonies from the aTC plates were selected and each resuspended in 20 μL PBS. 2 μL of this resuspension was used in a colony PCR reaction to assess genotype. Simultaneously, the remaining 18 μL of the PBS-resuspended sample was plated for single colonies on a TS + Erm plate. Using an isolated colony from the plate struck for single colonies, we resuspended a colony in 20 μL 1× PBS and used 2 μL of this resuspension in a colony PCR of the target region. Once the mutation was confirmed by a band shift on the electrophoresis gel, we seeded the remaining 18 μL of colony resuspension into a 40 mL TS broth culture to begin plasmid curing.

### 4.10 ∣ pGBSedit Plasmid Curing From Confirmed Mutants

40 mL TS broth cultures were inoculated using an isolated colony that has been genotypically confirmed and grown stationary at 37°C overnight. A second passage in 40 mL of TS broth was performed using 100 μL of the previous culture as inoculum. 1:10,000 and 1:100,000 dilutions of the grown second passage were plated on TS agar plates. Colonies were dual patched onto TS and TS + 5 μg/mL Erm agar plates to detect cured clones by antibiotic sensitivity profiling.

### 4.11 ∣ Outgrowth and Selection for Free End Joining Mutants

To use pGBSedit without an editing cassette to generate alternative end-joining mutants, the same experimental procedures are followed as with homology-driven mutagenesis. However, because free end joining mutants can be the result of indels too small for easy detection by comparing PCR bands on an electrophoresis gel, we first performed a colony PCR screen on candidate colonies, checking for the presence or absence of the wild type Cas12a target (the sequence complementary to the pGBSedit spacer). Colonies that screened negative were then further tested by PCR and sequencing to characterize indels.

### 4.12 ∣ CFU Quantification of Cas12a Selective Force and Mutagenesis Frequency

Individual TS + Erm cultures were inoculated using GBS harboring a targeting pGBSedit plasmid and grown for 8 h. Serial dilutions and CFU plating on TS + Erm plates for each overnight culture were performed to access total viable CFU/mL. Simultaneously, 100 μL of each overnight culture was plated onto TS + Erm + 500 ng/mL aTC at both 1:10 or 1:100 dilutions. After 36 h of growth, colonies on aTC plates were counted and compared to the starting culture colony counts. Colonies from the aTC plates were selected and screened for genotypes by NEB OneTaq colony PCR (Cat. M0482L). Genotypes were counted based on 1% agarose gel electrophoresis PCR band sizes or sequencing results.

### 4.13 ∣ Outgrowth and Induction of Knockdown Strains

GBS harboring the pGBScrispri plasmid are grown in TS + Erm to maintain the plasmid. For phenotypic assays, induction of knockdown was achieved by the addition of 250 ng/mL aTC (stock 2 mg/mL in water) to cultures from isolated single colonies. Cultures were grown stationary at 37°C for 18 h.

### 4.14 ∣ Fluorescence Assay

10 mL TS broth cultures were inoculated using isolated colonies and grown overnight at 37°C shaking. Overnight cultures were washed twice with PBS before being OD_600_ normalized to 1 in PBS. 10 mL of OD_600_ normalized cultures were spun at 4000 RPM for 5 min, then resuspended in 1 mL of PBS to 10× concentrate. 200 μL of each sample was loaded in technical triplicates into black optical bottom 96-well plates. GFP was excited at 488 nm, and emission was measured at 515 nm.

### 4.15 ∣ RNA Purification and qRT-PCR

10 mL TS + Erm cultures were inoculated using isolated colonies and grown to mid-log (OD_600_ = 0.6) before being split into two 5 mL cultures: untreated and 250 ng/mL aTC-supplemented. Cultures were incubated at 37°C for 1 h post treatment before being spun down and resuspended in 1 mL of RNAprotect Bacterial Reagent (Qiagen Catalogue: 76506). Bacteria were incubated in RNAprotect at room temperature for 5 min before being pelleted in microcentrifuge tubes at 15,000 RPM for 2 min. Excess RNAprotect was removed, and pellets were stored at −80°C until RNA extraction. RNA protected pellets were thawed on ice, and RNA was extracted using the Qiagen RNeasy Kit (Cat. 74104) per manufacturer protocol. RNA was eluted with warmed (95°C) ultra-pure water in place of elution buffer. DNA was removed with the Invitrogen DNA-free Kit (Cat. AM1906) per manufacturer protocol. cDNA was synthesized with the AppliedBiosystems High-Capacity cDNA Reverse Transcription Kit (Cat. 4374966) per manufacturer protocol. cDNA and reverse transcription negative controls were 1:10 diluted with ultra-pure water before 1 μL was used as template in 10 μL qPCR reactions. Bio Rad SsoAdvanced Universal SYBR Green Supermix (Cat. 1725272) was used for all qPCR reactions. Target gene mRNA measurements were normalized to the housekeeping gene sortase A (*srtA*) mRNA.

### 4.16 ∣ Hemolysis Assays

10 mL of heparanized human whole blood was spun down for 10 min at 2000 RPM, then washed repeatedly in 5 mL of Hank's buffered saline solution (HBSS). Packed red blood cells (PRBC) were spun for 10 min at 2000 RPM, and the supernatant was removed with a serological pipette. 500 μL of PRBC were added to 50 mL of HBSS to achieve a 1% PRBC solution. 10 mL cultures of GBS harboring the pGBScrispri plasmid were grown for 18 h with or without 250 ng/mL of aTC, then pelleted and resuspended in 10 mL of PBS. 10 mL resuspensions were OD_600_ normalized to 1.0, then 10 mL of OD_600_ normalized A909 cultures were concentrated 10× by pelleting and resuspending in 1 mL of PBS. 10/84 cultures were 1:10 diluted in PBS after OD normalization. Hemolysis assays were performed with 100 μL of PRBC mixed with 100 μL of bacterial suspension and incubated at 37°C for either 15 min (10/84) or 1 h (A909). Hemolysis assays were performed in 96 well V-bottom plates in technical triplicate. After hemolysis, V-bottom plates were spun down at 2000 RPM for 2 min, and supernatants (100 μL) were collected for each sample, and absorbance at 415 nm was used to measure released hemoglobin in the supernatant. Percent hemolysis was calculated relative to a 1% triton control.

### 4.17 ∣ Whole-Genome Sequencing, Scaffolding, and Analysis

Genomic DNA samples were prepared for whole-genome sequencing (WGS) using the Illumina DNA Prep tagmentation kit with IDT for Illumina Unique Dual Indexes. Sequencing was performed on an Illumina NextSeq2000 platform with a 300-cycle flow cell to generate paired-end reads (2 × 150 bp). A 1%–2% PhiX control library was included to ensure optimal base-calling accuracy. Initial processing—including demultiplexing, read trimming, and analytics—was performed using Illumina DRAGEN software (version 3.10.12) (Behera et al. 2024). Quality control included FastQC metrics evaluation.

Further processing and genomic assembly utilized a custom bio-informatics pipeline. Reads underwent stringent quality filtering, trimming, and duplicate grouping using BBTools. Filtered reads were aligned to the A909 GBS strain reference genome to create a high-confidence consensus sequence, requiring a minimum depth of 10 reads at consensus sites. Quality-filtered reads were then de novo assembled into contigs using SPAdes (Bankevich et al. 2012) in careful mode. Contigs were scaffolded onto the consensus reference using RagTag (Alonge et al. 2022), followed by patching to resolve scaffold gaps and inconsistencies. The primary scaffold from each assembly was extracted and annotated using the Prokaryotic Genome Annotation Pipeline (PGAP) (Tatusova et al. 2016). SNPs were determined by aligning mutant strain reads to assembled parent scaffolds with Bowtie 2 (Langmead and Salzberg 2012) and counting variants in the alignment using Geneious Prime SNP annotation, set to a cutoff of ≥ 75% of reads showing the nucleotide change. Pipeline scripts and dependencies were managed within a custom conda environment and run locally.

## 5 ∣ Public Resources Availability

The following plasmids for GBS Cas12a editing, CRISPRi, and complementation are available at Addgene.

**Table:** 

Addgene plasmid number	Plasmid name
223200	pGBSedit Sham
223201	pGBScrispri Sham
223202	pGBScomp High
223203	pGBScomp Low

## Supplementary Material

Supplemental Data

Additional supporting information can be found online in the Supporting Information section. **Data S1:** mmi70022-sup-0001-DataS1.pdf.

## Data Availability

The data that support the findings of this study are available from the corresponding author upon reasonable request.
